# Supplementary figures and images for: An assembly-free method of phylogeny reconstruction using short-read sequences from pooled samples without barcodes
Source: PLoS Comput Biol. 2021 Sep 13;17(9):e1008949. doi: 10.1371/journal.pcbi.1008949 (PMC8460051; doi:10.1371/journal.pcbi.1008949)

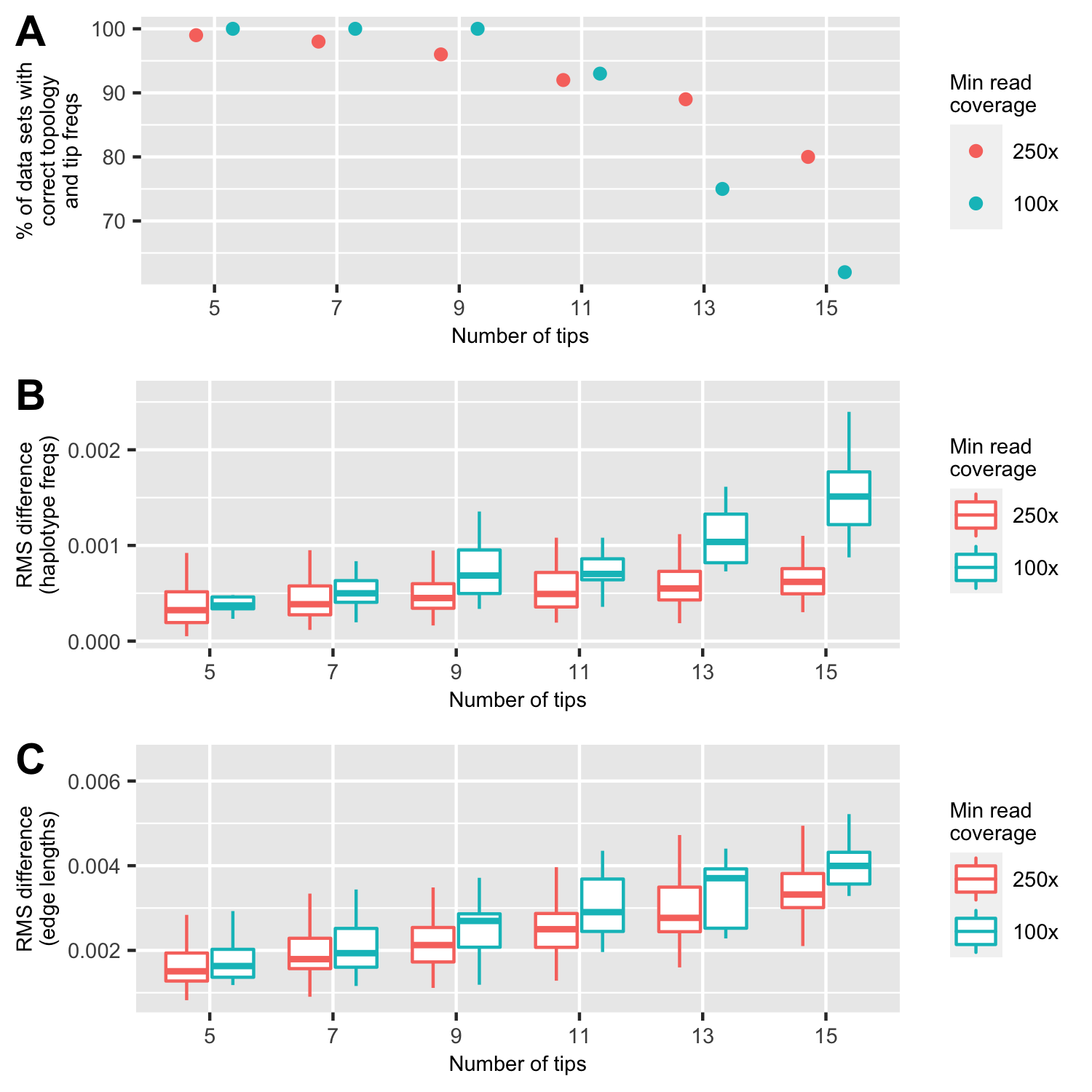

Supplement: S1 Fig — (A) Accuracy of AFPhyloMix between data sets of which the least abundant haplotype has at least 250x and 100x read coverages. (B) Root-mean-square differences between the actual and the predicted tip relative abundances for data sets with different read coverages. (C) Root-mean-square differences between the actual and the predicated edge lengths for data sets with different read coverages. (TIF) [file pcbi.1008949.s001.tif]

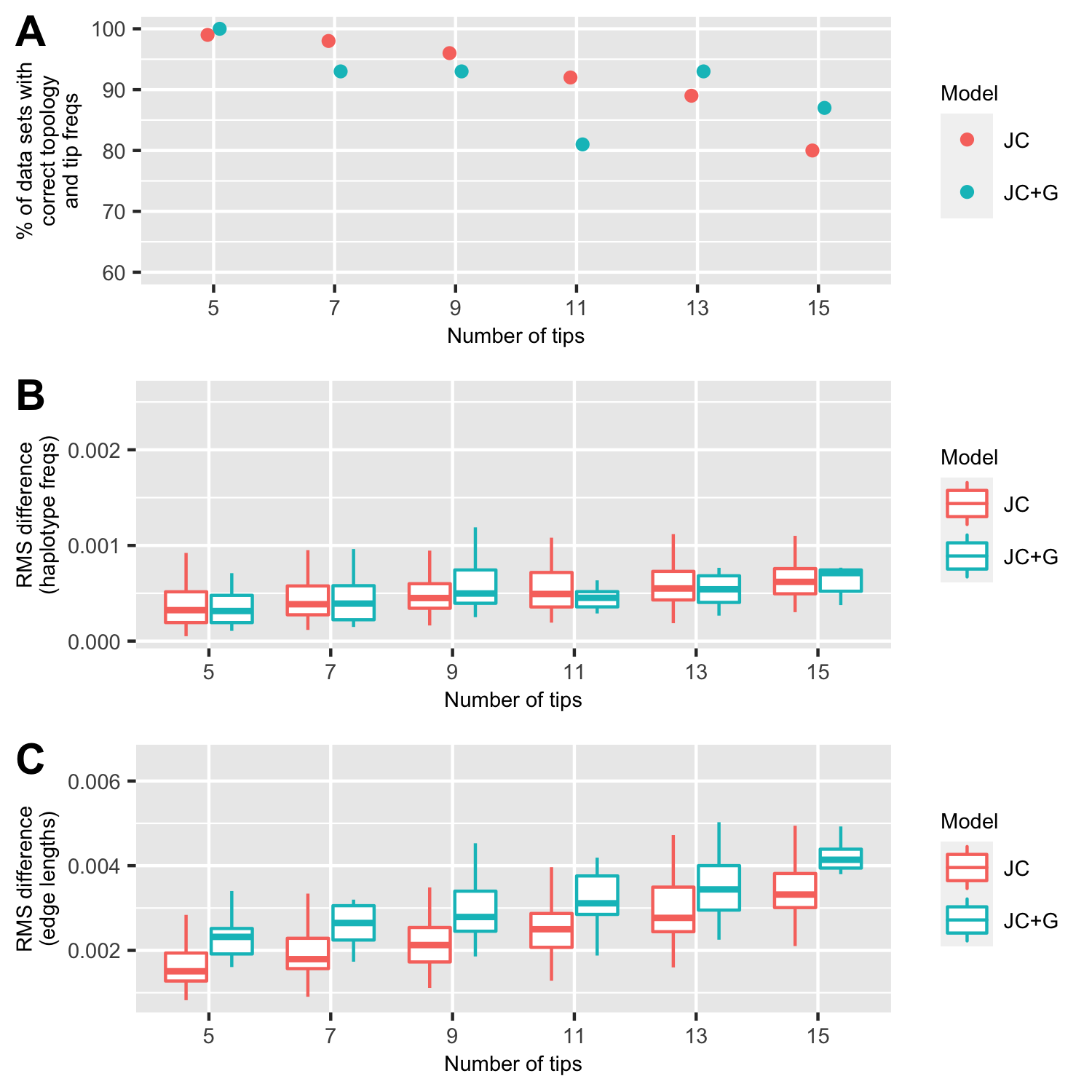

Supplement: S2 Fig — (A) Accuracy of AFPhyloMix between data sets evolved under a simple model—JC and a model with site variation—JC+G. (B) Root-mean-square differences between the actual and the predicted tip relative abundances for data sets evolved under JC and JC+G. (C) Root-mean-square differences between the actual and the predicated edge lengths for data sets evolved under JC and JC+G. (TIF) [file pcbi.1008949.s002.tif]
