# Supplementary material for: An assembly-free method of phylogeny reconstruction using short-read sequences from pooled samples without barcodes
Source: PLoS Comput Biol. 2021 Sep 13;17(9):e1008949. doi: 10.1371/journal.pcbi.1008949 (PMC8460051; doi:10.1371/journal.pcbi.1008949)
Supplement: S1 Text — (PDF) [file pcbi.1008949.s003.pdf]

Evaluation of AFPhyloMix on other simulated data sets

AFPhyloMix is expected to perform better under high read coverage. To examine the performance of AFPhyloMix under the situations with lower read coverage, we simulated another 96 data sets in which the least abundant haplotypes have at least 100x read coverage. The accuracy (regarding the topology, haplotype relative abundances, and edge lengths) drops reasonably when the coverage decreases (Fig S1). AFPhyloMix was also tested on another 96 data sets simulated under JC+G model (alpha=2), a model with site variations. The accuracy on this set of simulations was comparable to that obtained with a JC model without site variation (Fig S2). This result is expected, because the infinite-site model used in AFPhyloMix should also work under a JC model with site variations, as long as the situation is that multiple mutations seldom happen on the same site.

Fig S1. Performance of AFPhyloMix on simulated data with different read coverage. (A) Accuracy of AFPhyloMix between data sets of which the least abundant haplotype has at least 250x and 100x read coverages. (B) Root-mean-square differences between the actual and the predicted tip relative abundances for data sets with different read coverages. (C) Root-mean-square differences between the actual and the predicated edge lengths for data sets with different read coverages.

Fig S2. Performance of AFPhyloMix on simulated data evolved under two different substitution models. (A) Accuracy of AFPhyloMix between data sets evolved under a simple model - JC and a model with site variation - JC+G. (B) Root-mean-square differences between the actual and the predicted tip relative abundances for data sets evolved under JC and JC+G. (C) Root-mean-square differences between the actual and the predicated edge lengths for data sets evolved under JC and JC+G.
