# Supplementary material for: An assembly-free method of phylogeny reconstruction using short-read sequences from pooled samples without barcodes
Source: PLoS Comput Biol. 2021 Sep 13;17(9):e1008949. doi: 10.1371/journal.pcbi.1008949 (PMC8460051; doi:10.1371/journal.pcbi.1008949)
Supplement: S2 Text — (PDF) [file pcbi.1008949.s004.pdf]

Implementation details in Markov chain Monte Carlo

Moves in Markov chain Monte Carlo

AFPhyloMix applies the Metropolis algorithm and proposes changes in either tip relative abundances, error rate, or tree topology, serially. The following moves during the Markov chain Monte Carlo process are implemented. All these moves have Hastings ratio equal to 1.

- Update in tip relative abundances

  For every tip i on the tree, its relative abundance (f_i) is updated to $f_i + r$ where $r \sim U(-0.15, 0.15)$. If the new value of relative abundance is a negative value, then the relative abundance is $-(f_i + r)$.

- Update in the error rate

  The error rate (e) is updated to $e + r$ where $r \sim U(-0.0005, 0.0005)$. If the new value of error rate is a negative value, then the error rate is $-(e + r)$.

- Distribute the relative abundances between two tips

  Randomly select two tips i and j on the tree. Update their relative abundances f_i and f_j to r and $f_i + f_j - r$ where $r \sim U(0, f_i + f_j)$.

- Swap the relative abundances between two tips

  Randomly select two tips on the tree and swap their relative abundances.

- NNI

  Randomly select an internal edge on the tree and perform Nearest-neighbor interchange (NNI).

- Swap between two subtrees

  Randomly select two nodes (internal or terminal) A and B satisfying the following criteria: (1) A and B are not sister nodes; (2) A is not an ancestor of B; and (3) B is not an ancestor of A. Then swap between the subtree rooted at A and the subtree rooted at B.

- Merge and split

  Randomly select an internal node A with exactly two leaves i and j. Select another tip k which is not a sister node of A. If $f_k > f_i$, then use the following step to merge the leaves i and j and split the leaf k into two tips. First remove the tip i and j and turn A into a new tip with relative abundance $f_i + f_j$. Then add two children at the node k with relative abundances f_i and $f_k - f_i$.

- Combine move

  Among the moves of NNI, Swap between two subtrees, and, Merge and split, randomly select two of them and consider to perform the two moves together.

Prior distributions
The prior of haplotype relative abundance is a gamma distribution with rate parameter 0.1 and shape parameter 2. The prior of the tree topology is uniform across all the possible topologies, and the prior of the error rate a uniform distribution with maximum value of e_{max} (which is set to 0.01 for the reads produced from Illumina sequencing machines).

Metropolis-coupled Markov chain Monte Carlo
AFPhyloMix runs 8 Markov chain Monte Carlo processes in parallel: one cold chain and seven hot chains. The i-th hot chain's temperature is set to $(1 - i/8)$. A hot chain is randomly selected and its posterior probability is compared with that of the cold chain for every x iterations (where the value of x equals to the number of possible moves times 5, for example, $x = 12 \times 5 = 60$ for 5 haplotypes; while $x = 22 \times 5 = 110$ for 15 haplotypes). If the posterior probability of the hot chain is higher than that of the cold chain, two chains will be swapped. When swapping between two chains, we followed [1]'s implementation that the temperature of the two chains were exchanged instead of the states. Exchanging their temperatures are more efficient than exchanging their states, because the states which include many parameters are usually large in size.

References

1. Müller NF, Bouckaert RR. Adaptive parallel tempering for BEAST 2. bioRxiv. 2020;doi:10.1101/603514.
