# Supplementary material for: An assembly-free method of phylogeny reconstruction using short-read sequences from pooled samples without barcodes
Source: PLoS Comput Biol. 2021 Sep 13;17(9):e1008949. doi: 10.1371/journal.pcbi.1008949 (PMC8460051; doi:10.1371/journal.pcbi.1008949)
Supplement: S3 Text — (PDF) [file pcbi.1008949.s005.pdf]

To check the correctness of an estimated tree and report the minimum root-mean-square difference between the estimated and the actual haplotype relative abundances

SThe estimated tree is regarded as correct if the tips on an estimated tree can be paired up with the tips on the actual tree satisfying the following conditions:

1. the difference between the predicted tip relative abundance and the corresponding actual tip relative abundance paired with is less than 0.01; and
2. their unrooted topologies are the same.

A recursive algorithm has been implemented (1) to check whether two rooted trees, T_i with the root node i and T_j with the root node j, match each other in terms of their topologies and their tip relative abundances, and, (2) if T_i matches with T_j, to report the minimum sum-of-square differences between the estimated and the actual haplotype relative abundances. The idea is as follows:

1. if T_i and T_j do not have the same number of leaves, then T_i does not match T_j.
2. (base case) if both node i and node j do not have any children (i.e. they both are leaves), then they match each other if the difference between their tip relative abundances is less than or equal to 0.01.
3. (recursive) if both node i and node j have children, let i_l and i_r be the left and the right children of node i, and let j_l and j_r be the left and the right children of node j. Then T_i matches T_j if one of the following conditions is satisfied:
   (a) the sub-tree rooted at i_l matches the sub-tree rooted at j_l and the sub-tree rooted at i_r matches the sub-tree rooted at j_r; or
   (b) the sub-tree rooted at i_l matches the sub-tree rooted at j_r and the sub-tree rooted at i_r matches the sub-tree rooted at j_l

   If both the conditions (a) and (b) are satisfied, then the condition, which reports the minimum sum-of-square differences between the haplotype relative abundances, is selected.

The pseudocode of the recursive algorithm is shown in Algorithm 1. The recursive algorithm is designed for two rooted trees. We use the following procedure to reroot both the true tree and the estimated tree in order to check whether they have the same unrooted topology.
Let T_{true} be the true n-tip tree and T_{est} be the estimated tree reported by AFPhyloMix. We first relocate the root of the true tree to one of the terminal edges (i.e. the edges connecting to the leaves) arbitrarily, and let T'_{true} be the true tree after the relocation of the root. Then we try to root the estimated tree at every terminal edge. Let $T'_1, \cdots, T'_n$ be the corresponding n trees when placing the root of the estimated tree at different terminal edges. T_{est} is regarded as correct if one of these trees $T'_1, \cdots, T'_n$ matches T'_{true} according to the recursive algorithm.

Let ssd_{min} be the minimum value among all the sum-of-square differences between T_i' and T_{true}', for $1 \leq i \leq n$. The minimum root-mean-square difference (rms_{min}) between the estimated and the actual haplotype relative abundances is:

$$rms_{min} = \sqrt{\frac{ssd_{min}}{n}}$$

Algorithm 1: isMatch(T_i, T_j, ssd)

Result: return true or false, and ssd (i.e. Sum-of-Square Difference), where, $ssd = \infty$ if it returns false

$n_i \leftarrow$ number of leaves in T_i;
$n_j \leftarrow$ number of leaves in T_j;
$ssd \leftarrow \infty$;
if $n_i = n_j$ **then**
  if *both node i and node j are leaves* **then**
    $f_i \leftarrow$ tip relative abundance of node i;
    $f_j \leftarrow$ tip relative abundance of node j;
    if $| f_i - f_j | \leq 0.01$ **then**
      $ssd \leftarrow (f_i - f_j) * (f_i - f_j)$;
      return true;
    end
  else
    $i_l \leftarrow$ left child of node i;
    $i_r \leftarrow$ right child of node i;
    $j_l \leftarrow$ left child of node j;
    $j_r \leftarrow$ right child of node j;
    if *isMatch(i_l,j_l,ssd_1) and isMatch(i_r,j_r,ssd_2)* **then**
      $ssd \leftarrow ssd_1 + ssd_2$;
    end
    if *isMatch(i_l,j_r,ssd_1) and isMatch(i_r,j_l,ssd_2)* **then**
      if $ssd_1 + ssd_2 < ssd$ **then**
        $ssd \leftarrow ssd_1 + ssd_2$;
      end
    end
    if $ssd \neq \infty$ **then**
      return true;
    end
  end
end
return false;
