# Supplementary material for: An assembly-free method of phylogeny reconstruction using short-read sequences from pooled samples without barcodes
Source: PLoS Comput Biol. 2021 Sep 13;17(9):e1008949. doi: 10.1371/journal.pcbi.1008949 (PMC8460051; doi:10.1371/journal.pcbi.1008949)
Supplement: Attachment — Submitted filename: cover_letter.pdf [file pcbi.1008949.s006.pdf]

Australian National University

Dr Thomas K. F. Wong

Research School of Biology
College of Science
Australian National University
Action, ACT 2601 Australia

Prof Daniel Beard, Prof Joel O. Wertheim
PLOS Computational Biology

16th August 2021

Dear Prof Beard and Prof Wertheim,

Re: Revision of manuscript

Thank you for your email regarding our manuscript titled "**An assembly-free method of phylogeny reconstruction using short-read sequences from pooled samples without barcodes**" submitted for consideration as a research article in *PLOS Computational Biology* journal. We have addressed all the comments. Please find the details of the amendments as follows.

Responses to the reviewers' comments:

Reviewer 1: The authors present an interesting and novel approach to phylogeny reconstruction. I would like to see a few changes prior to publication, some textual, but I'd also like to see two more kinds of simulation study to clarify the behavior under certain conditions. Text changes:

1) Authors need to make it more prominently clear (maybe even in the abstract?) that this approach will estimate the tree and the haplotype frequencies, but not the actual sequence associated with each haplotype. This, along with the requirement that you must know, a priori, the number of haplotypes, is a rather severe limitation, and I struggle to think of a practical use for the proposed method. The typical case where we know the number of haplotypes in advance is when we explicitly mix a number of individually homogenous samples, but then we'd get a tree and frequencies, but have no way of saying which sample was associated to which tree tip, nor obtain a haplotype sequence for each sample. My particular concern is that, as it is currently written, a biologist may read the manuscript and not appreciate this limitation, design a sequencing experiment without barcodes, run the software, and then become immensely frustrated that they can't recover the haplotype sequences with each of their mixed components.

⇒ **Thanks for the suggestion. A sentence: "AFPhyloMix estimates the phylogeny of the haplotypes and their relative abundances, <u>without constructing the haplotype sequences</u>." has been added into the last paragraph of the introduction section, to explicitly inform the reader that this method only focuses on the estimation of phylogeny and haplotype frequencies, but not the construction of haplotype sequences.**

2) "We implemented MCMCMC in AFPhyloMix, which reports a the tree topology and the tip frequencies which gives the highest value among all the resulting posterior probabilities along the computation. "This sounds like you're reporting a single sample from the chain? This is not very Bayesian in spirit, and could suffer from high variance, possibly differing substantially from one run to another (far more so than usual approaches to summarize MCMC chains). Given that tips are not tied to sequences in the usual way, so the "identity" of a tip can just drift, un-anchored, during sampling, I can see that you might have difficulties using other approaches to extract a consensus tree from the chain, but your chosen approach feels like it requires a little more justification and clarification.

⇒ **Thanks for the comment. In fact, AFPhyloMix reports the sample every 100 rounds along the cold chain. However, as mentioned by the reviewer, due to the change of the tip frequencies along the chain, there is currently no general algorithm to construct the consensus of unlabelled trees that can be applied meaningfully to this problem. For the sake of simplicity, AFPhyloMix, therefore, also reports the tree topology and the tip frequencies which gives the highest value among all the resulting posterior probabilities along with the computation, and the performance of AFPhyloMix was then evaluated based on this tree topology and the tip frequencies. We have modified the manuscript so that it clearly mentions this.**

3) "During the experiments, the following modified formula was found to have a better accuracy on the estimation of the tree topology and the tip frequencies: "Could the authors explain this a little? How much better? Why did they try this formula in the first place? What is the explanation for its better performance?

⇒ **We have updated the manuscript accordingly to explain it more clearly. In fact, we adopt the modified formula because it allows MCMC to converge at a faster speed (instead of a better accuracy). Equation (6) which is the formula in the first place includes the parameters: tree topology, tip frequencies, sequencing error rates, and the probability of a mutation on each edge. The modified formula (i.e. Equation (7)) does not contain the probability of a mutation on each edge and only focuses on the tree topology, tip frequencies, and the error rate. Because of the fewer number of parameters needed to be estimated in the first step, we see that the MCMC chain converges at a faster rate, especially as the number of haplotypes increases.**

4) "Whereas it is theoretically possible to simultaneously infer edge lengths and topologies, we have found that this does not deliver accurate results. "How inaccurate were they? This seems like strange behavior.

⇒ **This question is related to the previous question. The manuscript has been updated to explain this more clearly. Whereas it is theoretically possible to simultaneously infer**

edge lengths and topologies, in preliminary simulations, we have found that this does not deliver accurate results. We think that this is because, in the absence of sequence information at the tips, inferred mutations will naturally favour long branches, thus lowering the probability of seeing short branches.

5) "The estimated result is regarded as correct if the tips on an estimated tree can be paired up with the tips on the actual tree satisfying the following conditions: (1) the difference between the predicted tip frequency and the corresponding actual tip frequency paired with is less than 0.01; and (2) their topologies are the same. "It isn't clear that this will produce a unique pairing of estimated to true tips, especially if the tip frequencies are similar (even at a single "cherry" - https://rdrr.io/cran/ape/man/cherry.html). If not, how do you efficiently choose the optimal tip-to-tip configuration to minimize later deviation estimates?

⇒ **We have listed out the details of the method, in the Appendix Section, for checking the correctness of an estimated tree and computing the minimum root-mean-square differences between the estimated and the actual haplotype frequencies. The method makes use of a recursive algorithm, which checks whether two rooted trees match each other in terms of their topologies and their tip frequencies. The method also reports a set of pairings between the estimated and the actual tips that give the minimum root-mean-square differences, among all the possible sets of pairings that lead to the valid matching between two trees. There may exist more than one set of pairings with the same minimum root-mean-square differences, but the ambiguity will not affect our results (like Figure 5) in the manuscript.**

6) Typo: "AFPhyloMix adopts a Bayesian model of inference to estimate[s] the phylogeny"
7) Possible typo: "To further examine the [derivation] between the predicted tip frequencies 328 and the actual sample concentrations" "deviation"?

⇒ **Thanks! The typos have been corrected.**

8) Simulation request
This approach might not be especially robust to violations of the underlying model. The simplest of these is where rates vary over sites. Standard phylogeny inference is quite robust to such variation, but something that relies on SNP frequency distributions in the manner described here may not be. I suggest two kinds of studies to investigate such model violations: A) Identical to your current simulation setup, but with varying levels of site to site variation in the Jukes Cantor mutation rate. B) Take a set of "real" haplotypes that have evolved through a real evolutionary process (e.g. a genome segment for a random set of mammals, or bacteria, or...), and use these as the basis of the simulation, where each simulation replicate involves the assignment of frequencies to each haplotype, and then the paired-end read simulation, etc. The "true" tree is the phylogeny inferred from the full haplotypes. I suggest finding a biological example where many genomes are available, so you can randomly select N each time, rather than using the same N over and over. Note: perfect performance in these cases shouldn't be a requirement for publication - I just think it should be well-characterized exactly how things break down in these scenarios.

⇒ **Thanks for the suggestions. Another 96 sets of data sets have been simulated under the JC+G model - a model with site variation. The data sets have been tested by AFPhyloMix and the experimental result has been added to the Appendix section. The accuracy was comparable to that with a JC model with no site variation of rates (Figure 10). The result is expected, because the infinite-site model used in AFPhyloMix should also work under a JC model with site variations, as long as the situation is that multiple mutations seldom happen on the same site. The analysis of some long genomic real data sets by using AFPhyloMix will be our future direction.**

Reviewer 2: This manuscript addresses a critical problem in working with sequence data - separating haplotypes that are all sequenced together and are difficult to separate through pre-processing. This new approach addresses this challenge and also identifies the frequency of each haplotype and the relationship among haplotypes. The manuscript does a nice job explaining the method, and then using both simulations and an empirical example to test the method. However, there are some important aspects of this paper that would benefit from further explanation and rewriting.

First, I can see very important applications of this method that could be added to the introduction to greatly improve the motivation for the work. Primarily, understanding haplotype frequencies and the phylogeny of haplotypes in a mixed sample would seem to benefit naturally mixed samples, such as coinfections (e.g. malaria or SARS CoV-2). Focusing in the introduction dealing with quantities of data is irrelevant, while concern about pre-processing time and cost seems secondary to being able to sort our coinfections to which there is currently no solution. Rewriting isn't strictly necessary but would provide stronger motivation for the research.

⇒ **We thank the reviewer for noting the value of the method for coinfections. We have added the following sentence (here in *italics*) to this paragraph in the introduction: "More importantly, there are some samples where barcoding is impractical. For instances, rapidly evolving viruses (e.g., Human Immunodeficiency Virus (HIV) and Hepatitis C Virus (HCV)) typically exist as a collection of genetically diverse genomes within an infected individual. *In some instances, hosts are infected with different strains of the same virus (i.e., coinfection).* To sequence one or more target genes from a collection of these viruses using barcoding, one would need to isolate individual viral genomes before library preparation. This can be done, but again, is time-consuming and laborious."**

The methods section begins with the expectations and calculations. However, this section then begins to explain some results (e.g. Figure 1C, 3B, ln 119+). The flow into the results is logical, except that the observed distribution is shown prior to any explanation of how it was generated. The simulations are not explained until the later section. I suggest significantly rearranging the Methods and Experiment sections so the theoretical expectations are provided first, then the simulations, then the results. Note that there are several other results integrated into methods (e.g. line 94, 212, 288) that should be moved as well.

Consider combining Fig 1B and C - overlaying the dots (expectation) on histogram (observation) would make the comparison clearer. Additionally, the points in 1B should be different shapes. B&C may need to be a separate figure in results. Similarly, Fig 3B is also results from simulations and may need to be moved, and overlaying A on B might make the comparison easier. The caption for Fig 1 should explain frequency v ratio so that that the caption can stand independently from the text.

⇒ **Thanks for the suggestion. The Methods and Experiment sections have been modified and the theoretical expectations are provided in the beginning, followed by the simulations and the results. Since there is much information in Figure 3B, keeping Figure 3A and Figure 3B separately seems to be clearer for the readers. To make the manuscript consistent, we have also separated Figure 1B and Figure 1C..**

I found the kangaroo data to be an odd example. It is clearly easy enough to tag these samples and get separate sequences for each sample. That said, it is an example that allows testing this method. To motivate its use better I suggest removing the discussion of kangaroo meat, which is completely irrelevant to AFPhyloMix, and just say this is a useful example where we could compare barcoded and not - you really could have picked any example where you mix samples at different concentrations.

⇒ **Yes, you are right that the main idea is to use real genomic sequences to evaluate AFPhyloMix and we could have picked any species for the experiment. Regarding the description of the arrangement of the Kangaroo meat, we suggest keeping it in because there may be an ethical concern about the source of the material.**

The terms ratio and frequency are challenging, primarily because the authors make statements like "the expected SNP ratio ... would be... which is the frequency of tip A" (line 84). Ratio and frequency mean very specific things in this paper so the use of those words should be avoided otherwise. Ratio (an individual SNP) depends on sample proportion while frequency (across SNPs) depends on branch length.

⇒ **Thanks for the suggestion. The terms "tip frequency" and "haplotype frequency" have been changed to "tip relative abundance" and "haplotype relative abundance".**

Simulations were done with unrealistically high coverage. It would be helpful to know how well this approach works with lower coverage.

⇒ **Another 96 data sets with lower read coverage have been simulated. AFPhyloMix has been run on this data set and, as expected, the accuracy (regarding the topology, haplotype relative abundances, and edge lengths) dropped reasonably when the coverage decreased (in Figure 9 in Appendix).**

Why is the root sequence used as the reference for alignment? This seems unrealistic. This choice should be justified or a reference should be chosen from the "extant taxa".

⇒ **As we note in the manuscript, our method works with haplotypes from within a population or species. There are many possible ways of choosing the reference**

sequence, but because the difference between the root sequence and those of the extant sequences is small (root-to-tip distance of 3%), we believe that the root sequence is a reasonable choice. Additionally using the root sequence as a reference sequence can provide us more control on the data, because we can make sure the similarities between the reference sequence and all other sequences are within a certain limit.

The abstract and introduction refer to amplicon sequencing, while the simulations are for small genomes. I suggest changing the abstract and introduction to match what was actually done.

⇒ **Thank for the suggestion, but we think the amplicon sequencing can be applied to both small and long genomes, as long as the amplicon length is longer than the read length.**

There are statements throughout such as Fig X shows... This phrasing is very awkward as it makes the results unclear. Please state actual results and refer the reader to the figure.

⇒ **Appreciate the suggestion. We think it is a matter of a different writing style. In order to have a better presentation of the results, some of the sentences have been updated accordingly.**

Fig 5B and C - consider normalizing these values. It's unclear what y-values are meaningful. Also, describe the quality of the results in the caption.

⇒ **The y-values represent the root-mean-square (RMS) differences between the predicted and the actual values. In other words, these values can be approximated as the average of the absolute differences between the estimated and the real values. In Figure 5B, we can see that all, with few exceptions, RMS differences are below 0.001, which means, on average, the estimated haplotype relative abundances are within ± 0.001 of the actual values. Similarly, in Figure 5C, as most of the RMS differences are below 0.004, the estimated edge lengths, on average, are within ± 0.004 of the actual values. Thanks for the suggestion. The description of the results has been added in the caption.**

There are some minor typos and odd word choices throughout that I hope will be caught on rereading / in proofs.

⇒ **The manuscript has been updated accordingly.**

Reviewer 3: Wong and colleagues present an interesting method to co-estimate the topology, branch lengths, tip frequencies and error rates (sequencing errors or others) from mapped reads, sequenced from a pooled DNA library, without barcoding for subsequent demultiplexing. As a population geneticists working on ancient DNA, I am a bit outsider to the field, and need a few additional (mostly minor, I think) clarifications:

1) the applicability of the method. Authors say in the introduction that: "More importantly, there are some samples where barcoding is impractical. For instances, rapidly evolving viruses(e.g., Human Immunodeficiency Virus (HIV) and Hepatitis C Virus (HCV)) typically exist

as a collection of genetically diverse genomes within an infected individual.". I truly understand your point here, but ...at the same time: "In our simulations, AFPhyloMix achieved at least 80% accuracy at recovering the phylogenies and frequencies of the constituent haplotypes, for mixtures with up to 15 haplotypes.". How many virus haplotypes do the authors expect to coexist during an infection? Probably way many more than 15, isn't it? How high is the mutation rate in viruses? Probably incompatible with an infinite sites model (?).How to justify then the use of AFPhyloMix? What is the typical problem then were we can apply AFPhyloMix? Only mtDNA, given the poor reference assemblies of Y chromosomes? How expensive it is to sequence 10-15 mitochondrial genomes with barcodes? Not much really. Why do we need AFPhyloMix then? I am playing the devils advocate to motivate the authors to better justify the biological problems that could be addressed with AFPhyloMix in the introduction (I am sure there are). At the end of the day, this is what will condition the utility and the impact of their program.

⇒ **This is an interesting point. With respect to viral genomes, early on in infection, we can expect a small number of haplotypes. This is particularly true if there is coinfection of two or more different strains of a virus.**

⇒ **The point about mtDNA is a good one, but since we are recovering relative abundances of the haplotypes, it is worth noting that we are not sequencing a max of 15 individuals, but rather a great many individuals, each of which may belong to one of the (max)15 haplotypes. So, in truth, what AFPhyloMix does is recover the haplotype frequency from many different individuals.**

2) the authors digest very well their equations making easy the reading and the understanding of the model, up until equation 6. They justify theoretically one equation, but suddenly "the following modified formula was found to have a better accuracy on the estimation of the tree topology and the tip frequencies", without much further explanation, despite equation 6 being only an approximation. Could the authors please elaborate this a bit more, including a non-mathematical (more intuitive) description of equation 6 and why (they think) it works better (no simulations shown proving this). It might be obvious, but I am bit lost there, and I suspect that other readers will be as well.

⇒ **We have updated the manuscript accordingly to explain it more clearly. The modified formula has fewer parameters which only includes the parameters of tree topology, tip frequencies, and the sequence error, but not the edge lengths. This allows MCMC to converge at a faster speed (instead of a better accuracy). Adopting the modified formula with fewer parameters allows MCMC to converge faster and can speed up the computation.**

3) what is the running time of the program? The authors discuss about the algorithm complexity, but a minimal description on the absolute scale would be informative. I guess AFPhyloMix is relatively fast given the number of simulations done, but readers might wonder if takes 1 week or more with e.g. 15 long haplotypes.

⇒ **Thanks for the suggestion. The average running time of AFPhyloMix on the simulated data sets have been added and are listed in the Table 1. It took around 4.5 days on a**

simulated data set with 15 long haplotypes on a machine with 4 x 16-core Intel Xeon 2.10GHz CPUs.

4) Why not using the information of the pair ends (if paired-end data is available) to help defining the haplotype? Imagine there are linked mutations at both reads of each pair. This would provide additional information on the read<->haplotype assignation, which could especially help refining estimates of the haplotype frequencies at the tips.

⇒ **This is a good point. In fact, we thought about considering the linked mutations at both reads of the same pair. For some data sets, the two reads of the same pair may overlap each other and, inside the overlapped region, some bases from one read may not match with those from another read. A possible strategy to deal with this is to consider only the positions with the same nucleotides from both reads. If so, then the error rate on the overlapped regions would be much lower than the error rate on the other regions, and the error model stated in equation 2 will not be correct. For the sake of simplicity, we do not consider the linked mutations at both reads of the same pair in this manuscript, but we may consider this by revising the error model in the future.**

5) What is the impact of the read length? In their empirical experiment, the authors test AFPhyloMix with reads that are 150bp-long and mtDNA , where the mutation rate is probably greater also in this species. I guess however that shorter reads and lower mutation rates reduce the chance of having more than one mutation per read, perhaps impacting the accuracy of the method. I am just asking whether the authors could clarify/elaborate a bit on this. Also, what is the substitution model or mutation rate used in their simulations?

⇒ **Thanks for the questions. Discussion about the impact of the short read length and the low SNP frequency (i.e. low mutation rate) has been added in the fourth paragraph in the Discussion Section. The substitution model is JC and the root-to-tip distance is 0.03, which means that the expected number of substitutions per site from root to tip is 3%.**

6) Is there any chance to add an independent function, once topology, branch length and tip frequencies are estimated (i.e. given the full phylogeny) to classify each read (or read pair) as belonging to one haplotype or another? That would be helpful for demultiplexing without barcodes. I understand this can be too much work for a revision, but I encourage the authors to think in implementing it after the publication (if the rest of concerns are satisfactorily addressed).
Thanks again for your efforts and congratulations for this nice approximation.

⇒ **Thanks for the suggestion and the encouragement. This is exactly the direction we have been working on now. We make use of the pair-end information here and compute the probability of each read pair belonging to one haplotype. Then we try to construct each haplotype sequence based on the read pairs classified to that haplotype.**

Thank you very much for your consideration of this manuscript.

Yours sincerely,

Thomas

Dr. Thomas K. F. Wong
Thomas.Wong@anu.edu.au
